# Salicine in Diarrhœa and Dysentery

**Published:** 1875-06

**Authors:** T. Curtis Smith

**Affiliations:** Middleport, O.


### SALICINE IN DIARRHŒA AND DYSENTERY.

BY T. CURTIS SMITH, M. D., MIDDLEPORT, O.

Remedies that are new often have a "run," more or less prolonged but in time they are proved to be of less real value than at first supposed; and they settle down to their proper level, among the thousand and one remedies or drugs mentioned in various medical works. The new use of an old remedy sometimes has a similar "run." But this is not as apt to be extensive, as in the care of an entirely new agent; but if my observation is correct, it is more apt to be substantial. Salicine has lately been brought forward in a new *role.* Will it stand the test of clinical experience? A writer here and there speaks of it with great favor. I will in a general way give my experience with the agent, and leave the readers of the RECORD to judge for themselves as to its merits.

My attention was first called to the agent as a remedy in diarrhœa, by Dr. J. B. Mattison, of Chester, N. J., in an article in the *Med. and Surg. Reporter*, afterwards by the same in the *Southern Medical Record.* Next I observed a brief report of its great value in the last named journal, from Dr. J. D. Tucker, of Jackson, Tenn., page 590. vol. iii, with special reference to its value in colliquative diarrhœa. While these papers made a sufficient impression on my mind to cause me to remember them, they were not sufficient to cause me to adopt them at the time, probably for the reason that I was controlling the disease quite fairly with other and more standard remedies.

In the summer of 1874, however, I met with quite a number of very obstinate and chronic cases, which resisted all the remedies I had yet prescribed for their relief. Upon relating the cases to a professional friend one day, (Dr. J. C. Bishop, of this place), he kindly, recalled to mind the papers on salicine, and made the statement that he was using it with perfect success in controlling the disease; even after other agents had either signally failed, or only afforded temporary relief. I confessed that I did not understand the *rationale* of the action of the agent, but would try it anyhow. Accordingly I placed a lady patient upon its use, who was then having chronic diarrhœa. The case had been very rebellious to all astringents, anodynes, alteratives, etc. In short, she was very feeble; greatly emaciated; and suffering from indigestion; passages very frequent. I gave her five grain doses of salicine every four hours. To my surprise she was a little better next day. This case continued to gradually improve, till at the end of two weeks there was complete relief from the troublesome and exhausting discharges. From this date onward I continued to use the agent in all cases of obstinate diarrhœa, with almost unvarying success.

I have found it especially valuable in those cases of long standing, and those attended with more than ordinary debility of the alimentary canal. Not less is it valuable, in fact, its most useful field seems to be among those cases of infantile diarrhœa that we so frequently meet with in the summer months. These little creatures, when afflicted with diarrhœa of a chronic type, are among the most pitiable cases we ever see. They are always greatly reduced and weak, and very especially is the digestive organs greatly enfeebled. Salicine seems to meet such cases, and that too with a promptness that is surprising. Of course the sensible practitioner need not be told of the necessity of looking after and removing the cause of the disease, when it is possible. But often when this is done with the greatest care, we are not always crowned with success in our efforts to afford relief to the little sufferers. But with the agent in question, I think I have met with a less number of failures than before its use.

In cases of chronic dysentery, it is also very valuable, though probably not to the same extent, as in diarrhœa. Yet it is among the best remedies I have found.

What is the rationale of its action? This is a question of no little import; for it especially becomes us to give a reason for the faith that is in us. I am not fully satisfied that my explanation is correct; but feel that it is so in some measure.

I think the chief or principal value of the agent lies in its tonic properties. It is not only a tonic, but it is unirritating, which is a point worth remembering. The toning effect that it seems to exert evidently spends most of its force upon the mucous membrane. But the agent is also a valuable general tonic, and no doubt is of some value in these cases, by virtue of the general strength it seems to bring to the system at large. A person with a stout, healthy, muscular and nervous system, is not as apt to be the subject of a chronic, or colliquative diarrhœa, as one who is debilitated, and one who has healthy and strong digestive organs is certainly less apt to suffer, than one whose digestive organs are more or less debilitated; and as the agent is found to be of more value in cases where great debility is present than in others, we naturally infer that some of its value is due to its tonic properties. Yet we cannot explain all of its virtues in diarrhœa in this way, for if it is only its tonic properties that render it of such great value, why do we not find cinchona, columbo, quassia, cascarilla, etc., of equal value in restraining the discharges? We know that while these are sometimes valuable in such cases, they are far from being as uniformly so as the salicine. From this course of reasoning, based upon clinical facts, we are led to infer, unavoidably, that salicine has some other influence over the alimentary tract, than simply that of a tonic. That action must be alterative in character. That it is not simply astringent, I am fully persuaded, for it may be given freely, and long continued, to patients after recovery from diarrhœa, and it will not render them costive.—Neither will it produce constipation, when given for other purposes than that of controlling diarrhœa. Its effects in this trouble cannot be attributable to its antimalarial power; for it will far surpass the cinchona salts in controlling the discharges, and it is too well known, to even need mention, that these salts rank the highest amongst the antimalarial agents. In fact I have knwn salicine to check completely an obstinate diarrhœa, where quinine has been previously given without any apparent effect in controlling the frequency, or amount of the discharges.

It may be that a part of its salutary effects are exerted through its influence over the nervous system. I am very well satisfied that this system has more influence over the production and continuance of diarrhœa, through morbid impressions made upon it; than we are often wont to believe.

Again let it be remembered, that salicine is most valuable in colliquative and chronic diarrhœa, where there is great debility of the general system, and especially of the digestive tract, while it is of less value in acute cases; but is by no means to be despised in this latter class of cases.

The dose varies, of course, with age. I usually give an infant of one year or under, a grain every two to four hours, increasing the dose proportionately for older ones. For an adult five to ten grains will be proper. It is among the most harmless agents we possess, and little fear of giving too much need be entertained. Let me say, in closing, that I do not pretend that the agent will cure every case; or that it will relieve any case in the face of unremoved causes, that have excited the discharges. Common sense will dictate to the medical attendant the necessity of looking after these, as it will also tell him that all medicines are but secondary agents in effecting recovery. It is always necessary to look after the diet, water, location, and habits of the patient, and of the very air that he breathes, in order to assure success in the shortest possible time, and with least possible use of medicines, whatever those medicines may be.

